# Preimplantation genetic testing might not be the necessity for male patients with 47,XYY syndrome: A pilot study

**DOI:** 10.1002/rmb2.12650

**Published:** 2025-04-22

**Authors:** Fan Dong, Zhong Zheng, Ying Ding, Yi Ma, Si‐Qi Wang, Xiang‐Feng Chen, Ping Ping

**Affiliations:** ^1^ Department of Reproductive Medicine, Ren Ji Hospital Shanghai Jiao Tong University School of Medicine Shanghai China; ^2^ Shanghai Key Laboratory for Assisted Reproduction and Reproductive Genetics Shanghai China; ^3^ Shanghai Human Sperm Bank, Ren Ji Hospital Shanghai Jiao Tong University School of Medicine Shanghai China

**Keywords:** 47,XYY syndrome, IVF/ICSI, neonatal outcomes, pregnancy outcomes, preimplantation genetic testing

## Abstract

**Purpose:**

This pilot study aimed to explore the necessity for 47,XYY syndrome males (couples) to perform PGT rather than conventional In Vitro Fertilization (IVF)/Intracytoplasmic Sperm Injection (ICSI) cycles.

**Methods:**

A retrospective cohort study was conducted with 36 nonmosaic and mosaic 47,XYY syndrome patients (couples) undergoing 43 oocyte retrieval cycles (37 planned for PGT and 6 for IVF/ICSI) between December 2017 and December 2023. The couples were given either next‐generation sequencing‐based PGT or conventional IVF/ICSI followed by 45 embryo transfer (ET) cycles (38 from PGT and 7 from IVF/ICSI). The detailed cytogenetic results of the 129 embryos from PGT were analyzed, and the pregnancy and neonatal outcomes between PGT‐ET and conventional IVF/ICSI‐ET cycles were compared.

**Results:**

The PGT results showed that the chance of sex chromosome abnormalities was low (1.55%), with chromosomal errors being observed more often in autosomes. Importantly, no differences were observed in the rates of biochemical pregnancy, implantation, clinical pregnancy, ongoing pregnancy, pregnancy loss, live birth, and preterm delivery between PGT‐ET cycles and conventional IVF/ICSI‐ET cycles. Comparable results regarding gestational age, birthweight, low birthweight rate, macrosomia rate, male rate, as well as the rate of congenital anomalies were also observed between the two groups.

**Conclusions:**

Preimplantation genetic testing might not be necessary to conduct for 47,XYY syndrome males unless there are other indications. Studies with large populations are in demand to confirm the present results.

## Graphical abstract

Preimplantation genetic testing (PGT) results showed that the chance of sex chromosome abnormalities was low in 47,XYY patients (couples). Comparable pregnancy and newborn results were observed between PGT‐ET cycles and conventional IVF/ICSI‐ET cycles of 47,XYY patients (couples). Therefore, PGT might not need to be conducted for 47,XYY syndrome males unless there are other indications.

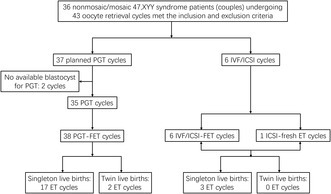

## 1 INTRODUCTION

The Y chromosome contains the sex‐determining region Y (SRY) gene of the males^1^ and is important for testicular development and maintenance of male fertility.^2^ The 47,XYY syndrome, also known as Jacobs syndrome^3^ or sometimes called “supermale”,^4^ is characterized by double Y chromosomes in the karyotypes of males and could be either nonmosaic (around 85.1%) or mosaic (around 10.6%).^5^ Its incidence rate was low (around 0.1% of males^6^), but it was more often observed in infertile males.^7^ The etiology of 47,XYY syndrome could be attributed to the YY disomy sperm, which was due to a meiosis II error of the sex chromosome.^8^

The fertility of males with 47,XYY syndrome is different among patients, and some patients are normozoospermic, whereas others suffer from oligoasthenozoospermia or even azoospermia.^9^ Although the fertility of 47,XYY syndrome males is diverse, previous studies have pointed out that an extra Y chromosome might affect male fertility in several aspects. First, Wong et al. reported that the level of spermatogenic cell mosaicism and their meiotic sex chromosome configurations might have an impact on the aneuploidy rate of 47,XYY patients' sperm, thus affecting their fertility status.^10^ Additionally, for the azoospermic 47,XYY patient, the process of meiotic prophase I was impaired, with significantly reduced pachytene spermatocytes.^11^ Moreover, the case of 46,YY sperm observed in 47,XYY syndrome might be attributed to a normal first meiotic division but an impaired second meiotic division.^12^ For the infertile 47,XYY patients, assisted reproductive technology (ART) has been widely used for them to have biological offspring.^13^, ^14^

Since that 47,XYY syndrome belongs to a form of aneuploidy, concerns have been raised about the risk of aneuploidy as well as other types of chromosome abnormalities in the embryos derived from such patients seeking ART treatments.^15^ But up to now, limited reports have given the results of the ploidy status of embryos from 47,XYY patients (couples).^15^, ^16^, ^17^, ^18^, ^19^ Among those studies, the majority were case reports or involved no more than five patients,^15^, ^16^, ^18^, ^19^ whereas the rest, although giving results for 51 patients, only focused on sex chromosome ploidy(autosome was not detected), and even so, it failed to give detailed information about what type of abnormalities occurred in sex chromosomes of the embryos.^17^ More importantly, all of those previous studies were based on fluorescence in situ hybridization (FISH); thus, they were unable to provide full information on the distribution of abnormalities in all chromosomes of embryos and were unable to give results on some detailed information of chromosome abnormalities, including segmental aneuploidy and mosaic levels. So to speak, the ploidy status and the distribution of chromosomal aberrations were largely unknown in embryos of 47,XYY patients (couples) receiving ART treatments. With the development of advanced detection technologies of embryos, especially next‐generation sequencing (NGS),^20^ it is far easier and essential to reveal the detailed embryo aneuploid status and distributions of embryo chromosomal abnormalities for 47,XYY patients in order to offer better ART strategies.

Preimplantation genetic testing (PGT) could identify genetically normal embryos for transferring to enhance pregnancy outcomes in ART^21^; thus, it is extensively used in couples with chromosomal abnormalities.^22^ Consequently, it is reasonable to conduct PGT in infertile males with 47,XYY syndrome, owing to its sex chromosomal errors. Some scientists also suggested that PGT might be used in ART for 47,XYY patients.^15^, ^17^, ^23^ However, whether PGT could really improve the clinical outcomes of 47,XYY patients is actually unclear because the above‐mentioned suggestions were mainly based on clinical experience and logical inference or based on results from insufficient sample sizes without appropriate controls for comparison. So far, there has been no study to discuss the necessity and superiority of PGT for 47,XYY males by directly comparing the PGT results and conventional In Vitro Fertilization (IVF)/Intracytoplasmic Sperm Injection (ICSI) results of these patients.

In the current pilot study, by analyzing the data of 36 nonmosaic and mosaic 47,XYY patients (couples) undergoing 43 oocyte retrieval cycles, we showed the PGT results of these patients, including detailed ploidy status and distributions of chromosomal abnormalities of the tested embryos. Moreover, we first compared the pregnancy and neonatal outcomes derived from PGT or conventional IVF/ICSI treatments of these 47,XYY syndrome patients. We hope the present study could help to reach a consensus on whether PGT should be recommended for males with 47,XYY syndrome.

## 2 MATERIALS AND METHODS

### 2.1 Study population

**FIGURE 1:**
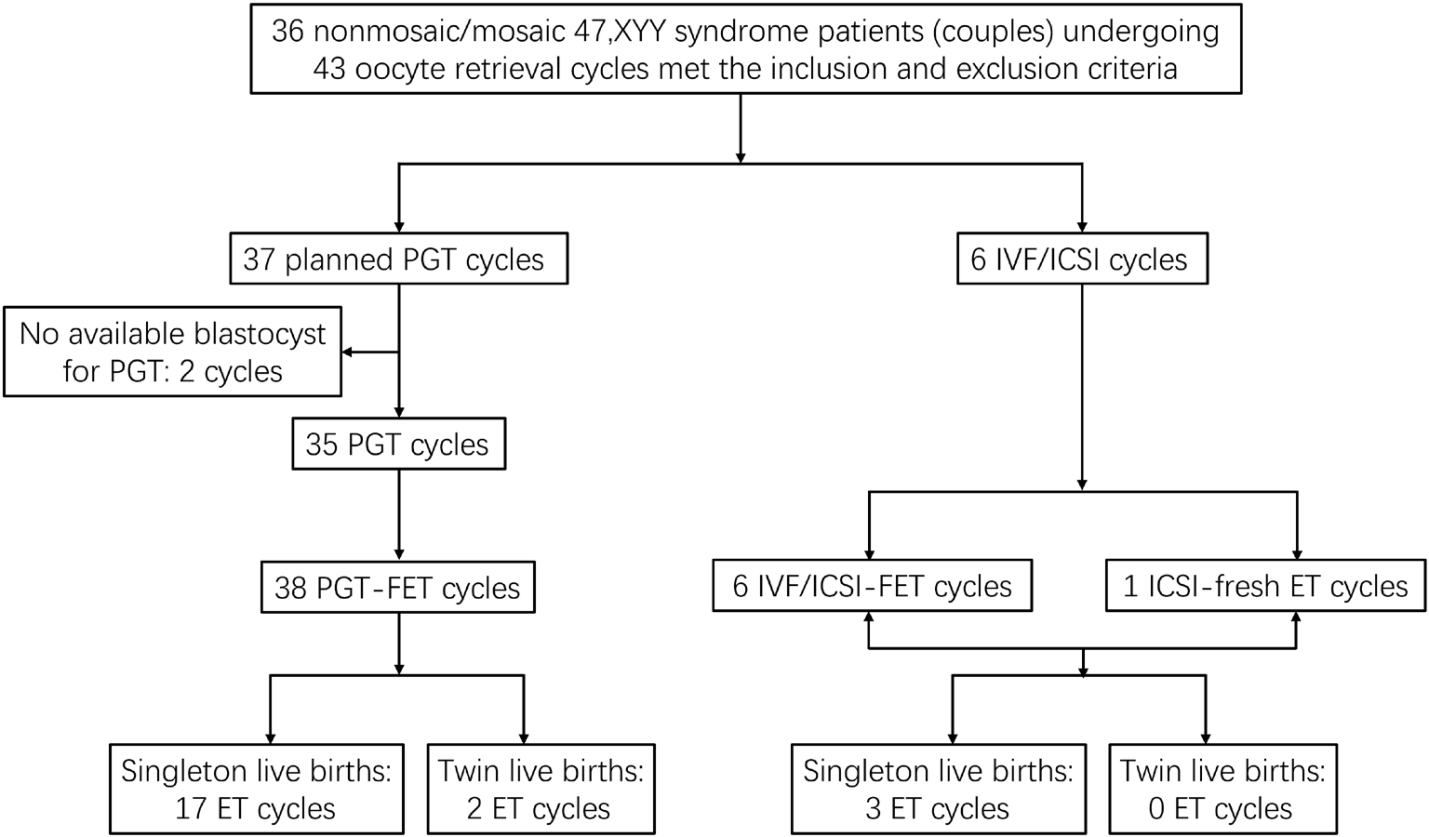
The workflow related to the enrolled patients and cycles in this study. ET, embryo transfer; FET, frozen embryo transfer; ICSI, intracytoplasmic sperm injection; IVF, in vitro fertilization; PGT, preimplantation genetic testing. By the last follow‐up time, three clinical pregnancies (two PGT and one IVF) have not reached live birth or pregnancy loss.

A total of 36 nonmosaic or mosaic 47,XYY syndrome male patients (couples) who received PGT or conventional IVF/ICSI treatment from December 2017 to December 2023 (according to female's oocyte retrieval date) in our center were included in the current study. The exclusion criteria for couples or cycles were (1) abnormal female karyotype (not including chromosome polymorphism), (2) oocyte retrieval cycles not within the above‐mentioned searching time frame, (3) oocyte retrieval cycles using donor sperm, and (4) canceled oocyte retrieval cycles or cycles with no oocyte retrieved. Considering the rarity of this disease (especially those patients receiving ART treatment), for couples that received more than one cycle in our center, we included all oocyte retrieval cycles that met the criteria. The data of couples were collected from the electronic medical record system or the original paper archive. The study workflow is shown in Figure 1. Among the 36 couples, 30 were planned for PGT, whereas 6 were planned for IVF/ICSI. In all, 43 oocyte retrieval cycles of these 36 patients (couples) were enrolled and analyzed.

For the diagnosis of 47,XYY syndrome, G‐binding chromosome analysis using peripheral blood was conducted on the male patients. All male patients performed routine semen analysis to evaluate their basal sperm level before the beginning of ART according to WHO Laboratory Manual for the Examination and Processing of Human Semen (WHO, 5th edition).^24^ The basal sperm level was classified as normozoospermia: volume ≥1.5 mL, concentration ≥ 15 × 10^6^/ml, total sperm count ≥ 39 × 10^6^/ejaculate, PR (progressive motility) ≥ 32%, total motility ≥ 40%; oligoasthenozoospermia: concentration < 15 × 10^6^/ml or/and total sperm count<39 × 10^6^/ejaculate or/and PR < 32%; severe oligoasthenozoospermia: concentration<5 × 10^6^/ml or/and PR < 10% (if conditions were met, the patient will not be double counted as oligoasthenozoospermia); and azoospermia: absence of sperm after semen centrifugation. For each female patient, the presence of a normal karyotype (or chromosome polymorphism) was verified by G‐binding chromosome analysis. All patients had completed comprehensive consultation and routine fertility assessment before the beginning of ovarian stimulation.

### 2.2 ART procedure

ICSI was conducted for the fertilization of all planned PGT cycles. Besides, the 6 couples that went through conventional IVF/ICSI treatments did not have PGT performed, based on physician's evaluation and patient's choice. The standard ART protocols of our reproductive center, such as ovarian stimulation, oocyte retrieval as well as laboratory procedures were previously described.^25^, ^26^ In brief, the ovarian stimulation regimen was individually designed with medicines, such as gonadotrophin‐releasing hormone (GnRH) analogs/GnRH antagonists plus gonadotrophins, contingent on the female's situation, and physician's recommendation. In 31 male patients with sperm in the ejaculate (27 planned for PGT and 4 for IVF/ICSI), a fresh semen sample was collected by masturbation on the day of oocyte retrieval. For the five azoospermic patients (3 planned for PGT and 2 for ICSI), testicular sperm was successfully retrieved in all cases by microdissection testicular sperm extraction (mTESE) surgery in our center, and four of them chose sperm cryopreservation in advance of ovarian stimulation, with frozen–thawed sperm used for fertilization. One patient initially chose testicular sperm aspiration (TESA) on oocyte retrieval day, but no sperm was obtained. Therefore, the oocytes of his wife were cryopreserved and thawed on the same day of his successful mTESE surgery. The retrieved oocytes were then fertilized by conventional IVF or ICSI^27^, ^28^ (MII oocytes was used for ICSI), and fertilization was assessed 17–20 h postinsemination. Oocytes were considered normally fertilized when two distinct pronuclei (2PN) were observed. Fertilization rate (2PN) was calculated as the number of normally fertilized oocytes divided by the number of oocytes used for insemination (IVF) or the number of MII oocytes (ICSI). Abnormal fertilization was defined as the observation of single or three or more pronuclei.^29^ Embryos were then cultured in vitro, and their cleavage was evaluated on day 2 and day 3. Only normally fertilized oocytes were used to calculate the cleavage rate of embryos. The day 3 (D3) good‐quality embryos were those normally fertilized embryos with 6–10 cells, ≤20% fragmentation, and (almost) equally sized blastomeres. In the planned PGT cycles, embryos were cultured to the blastocyst stage for cryopreservation and testing. In conventional IVF/ICSI cycles, apart from blastocysts, good‐quality cleavage embryos were also considered for transfer or cryopreservation. The blastocysts were evaluated according to Gardner criteria^30^ and both total blastocysts and good‐quality blastocysts were counted.^31^ Those day 5 or day 6 blastocysts that reached at least stage 3 and grade A/B of the inner cell mass and trophectoderm were considered good‐quality blastocysts.^31^, ^32^ As a note, in order to obtain viable blastocysts as many as possible, in our center, not only 2PN fertilized D3 embryos (but also some other embryos such as 0PN cleavage embryos) were cultured for blastocysts. Therefore, total/good‐quality blastocyst formation rates were calculated among D3 embryos that were actually used for blastocyst culture.

### 2.3 PGT procedure

For PGT cycles, blastocyst trophectoderm biopsy was conducted on day 5 or day 6 of embryo culture, followed by embryo cryopreservation. Afterward, whole‐genome amplification (REPLI‐g Single Cell Kit, Qiagen) was carried out.^33^ After establishing sequencing libraries (VeriSeq PGS Library Prep Kit, Illumina), NGS was conducted in line with protocols of the sequencing platform. The results were aligned to GRCh37/hg19 human reference genome. The segmental abnormalities of chromosomes were also confirmed as previously described with an optimal effective resolution of 4 Mb.^33^ Specifically, one patient, besides presenting the 47,XYY karyotype, had a pathogenic variant in the PKD1 gene (polycystic kidney), and thus performed PGT for monogenic diseases (PGT‐M) (the ploidy condition and segmental abnormality condition of chromosomes were also tested). The PGT‐M case was exceptionally based on single‐nucleotide polymorphism array (SNP array).

Ploidy condition was described as “Euploid” (aneuploid proportion < 30%), “Mosaic” (aneuploid proportion 30–80%) or “Aneuploid” (aneuploid proportion > 80%). Mosaicism referred to the simultaneous appearance of two or more genetically different cell lines in an embryo.^34^ Whole chromosomal aneuploidies referred to cells gaining or losing one or more entire chromosomes, while segmental aneuploidies referred to the gain or loss of the size of a chromosome.^35^ Sex chromosome abnormality was defined as the aneuploidy or mosaicism of sex chromosomes. The rate of euploid blastocysts was defined as the number of euploid blastocysts divided by the number of blastocysts receiving PGT. In accordance with the previous study,^34^ we defined the blastocyst as a whole aneuploid blastocyst as long as at least one whole chromosomal aneuploidy was found, and segmental aneuploid blastocyst or mosaic blastocyst was similarly defined. The total/whole/segmental aneuploid blastocyst rates were the number of total/whole/segmental aneuploid blastocysts, respectively, divided by the number of blastocysts receiving PGT. For mosaic rates, total mosaic blastocyst rate (the embryo only needs to be with mosaicism, regardless of the karyotype of each part) and mosaic euploid/aneuploid blastocyst rate (the embryo has to be a mosaic of euploid with aneuploid) were both calculated, as mosaic euploidy/aneuploidy could also have a chance to achieve normal live births.^36^, ^37^

### 2.4 Embryo transfer cycles

The regimens used for endometrial preparation in these couples were natural regimen and programmed regimen. The endometrial thickness was measured by ultrasound, and luteal phase support was offered as previously described.^25^ For most patients that had transferable embryos, frozen embryo transfer (FET) cycles were conducted (including all PGT cases). Only one couple (one cycle), who had their frozen oocytes thawed simultaneously with mTESE surgery (as above‐mentioned), received fresh embryo transfer. For PGT‐FET cycles, only one blastocyst was transferred after the application of progesterone. For IVF/ICSI‐ET cycles, no more than two cleavage embryos or blastocysts were transferred. In terms of embryo selection, in PGT‐ET cycles, both cytogenetic and morphological parameters were considered; in conventional IVF/ICSI‐ET cycles, embryo morphology was the only selection criterion followed. For pregnant females, the luteal phase support was continued until around 8–12 weeks of gestation.

### 2.5 Pregnancy and neonatal outcomes

Apart from the results of detailed ploidy status of PGT embryos, the measured outcomes of this study also included pregnancy and neonatal outcomes. In terms of pregnancy outcomes, biochemical pregnancy was defined as the positive pregnancy test (serum β‐hCG level ≥ 10 mIU/ml) around 14 days (or more) after embryo transfer^38^; implantation rate referred to the number of intrauterine gestational sacs detected by ultrasound per number of embryos transferred; clinical pregnancy was defined as the detection of the gestational sac by the transvaginal ultrasound scan around 4–5 weeks after embryo transfer^25^; ongoing pregnancy was defined as the viable pregnancy (fetus with heartbeat) that continued beyond 12 weeks of gestation.^25^ Biochemical pregnancy loss was defined as the observation of a biochemical pregnancy failing to progress to a clinical pregnancy,^39^ and clinical pregnancy loss was defined as the loss of a clinically recognized intrauterine pregnancy occurring throughout pregnancy. Of note, the spontaneous pregnancy reduction of a multiple pregnancy (the spontaneous loss of cardiac activity of one fetus^40^) was also considered one type of clinical pregnancy loss. Live birth was defined as the delivery of a living newborn (or living newborns) ≥28 weeks of gestation.^41^ Childbirth of twins was counted as one live birth.^42^ Preterm delivery was defined as the delivery of a fetus <37 weeks and >28 weeks of gestational age.^41^ In the current study, each pregnancy was followed up to either live birth/pregnancy loss or to the last follow‐up time (Jan 2024). For the record, the biochemical/clinical/ongoing pregnancy rate and live birth rate were calculated as per embryo transfer cycle, due to the fact that the incidence rate of 47,XYY syndrome is quite low and the number of patients is limited; hence, we enrolled all the ET cycles of each couple to maximize the available data. The clinical pregnancy loss rate and preterm delivery rate were calculated among clinical pregnancies, which was identical to previous studies.^38^, ^41^

As for neonatal outcomes, low birth weight was defined as the weight at birth of a newborn less than 2500 g, while macrosomia was the weight at birth ≥4000 g.^43^ Congenital anomaly was defined as the structural or functional anomalies that happened during intrauterine life, including both major (such as atrial septal defect) and minor (such as patent foramen ovale) anomalies.^38^ The rates in neonatal outcomes were calculated per newborn.

### 2.6 Statistical analysis

Continuous variables were reported as mean ± standard deviation (SD). Discrete and categorical variables were reported as frequencies (*n*) and percentage (%). For between‐group comparisons of continuous variables, the Mann–Whitney U test was employed. Chi‐square test or Fisher's exact test (as appropriate) was conducted to compare the between‐group difference of categorical variables. Statistical analysis was performed with GraphPad Prism version 9.2.0 (GraphPad Software, LLC). Fisher's exact tests for non‐2 × 2 contingency tables were calculated with the fisher.test function in R studio version 4.3.2 (Posit Software, PBC). Two‐sided *p* < 0.05 was considered.

## 3 RESULTS

### 3.1 Baseline features, ART process, and embryo results

A total of 36 couples underwent 43 oocyte retrieval cycles followed by 45 embryo transfer cycles that were included in the final analysis (Figure 1). The fertility of the male patients varied, and 12 patients had normozoospermia. Two‐thirds of the patients presented oligoasthenozoospermia (7 patients), severe oligoasthenozoospermia (12 patients), and azoospermia (5 patients). Among them, 30 couples were planned for PGT (grouped as planned PGT couples), whereas 6 were given conventional IVF/ICSI (grouped as IVF/ICSI couples). Of the 36 couples, 31 used ejaculated sperm (27 planned PGT and 4 IVF/ICSI couples) and 5 used testicular sperm (3 planned PGT and 2 ICSI couples). Of the 43 oocyte retrieval cycles (37 planned PGT and 5 IVF/ICSI), there were 45 ET (38 PGT‐ET and 7 IVF/ICSI‐ET). The average height of the male patients was over 181 cm. The range of female age was 23–44 years old. The mean maternal and paternal ages were both similar between planned PGT couples and IVF/ICSI couples. Besides, the other baseline features of couples, such as basal hormone levels and some infertility factors of female patients, were also comparable between the two groups (Table 1).

**TABLE 1:** Baseline features of the included couples.

Variables	All	Group		*p* Values
		Planned PGT	IVF/ICSI	
Couples (*n*)	36	30	6	
Female				
Maternal age (years)	30.00 ± 4.48	30.30 ± 4.64	28.50 ± 3.51	0.4992
Female BMI	23.32 ± 3.06	23.52 ± 3.13	22.34 ± 2.68	0.6875
Duration of infertility (years)	2.83 ± 1.89	2.75 ± 1.65	3.25 ± 2.96	0.8904
Prior gravidity (*n*, %)	7 (19.44)	6 (20.00)	1 (16.67)	>0.9999
Prior parity (*n*, %)	1 (2.78)	1 (3.33)	0 (0.00)	>0.9999
History of ART failure (*n*, %)	5 (13.89)	3 (10.00)	2 (33.33)	0.1858
AMH (ng/ml)	4.40 ± 2.41	4.34 ± 2.33	4.72 ± 3.02	0.7205
TSH (μIU/ml)	2.32 ± 1.26	2.37 ± 1.33	2.06 ± 0.80	0.6986
Basal FSH level (IU/L)^a^	6.30 ± 1.98	6.10 ± 1.56	7.52 ± 3.67	0.4837
Basal LH level (IU/L)^b^	4.99 ± 2.45	4.72 ± 1.83	6.33 ± 4.47	0.3888
Basal estradiol level (pg/ml)^a^	53.81 ± 56.33	57.68 ± 61.01	35.12 ± 15.02	0.5858
Basal prolactin level (ng/ml)^a^	22.98 ± 12.11	23.42 ± 12.85	20.67 ± 7.72	>0.9999
Basal testosterone level (nmol/L)^a^	1.01 ± 0.49	1.06 ± 0.50	0.74 ± 0.35	0.2012
Basal progesterone level (ng/ml)^a^, ^c^	0.36 ± 0.32	0.39 ± 0.35	0.28 ± 0.10	0.7131
Tubal factor (*n*, %)	8 (22.22)	6 (20.00)	2 (33.33)	0.5963
Diminished ovarian reserve (*n*, %)	4 (11.11)	3 (10.00)	1 (16.67)	0.5348
Endometriosis (*n*, %)	2 (5.56)	2 (6.67)	0 (0.00)	>0.9999
PCOS/PCOM on ultrasound (*n*, %)	10 (27.78)	8 (26.67)	2 (33.33)	>0.9999
Male				
Paternal age (years)	32.33 ± 5.41	31.77 ± 4.85	35.17 ± 7.55	0.3119
Male height (cm)	181.35 ± 6.74	181.78 ± 7.07	179.17 ± 4.58	0.2116
Male BMI	26.82 ± 3.64	27.05 ± 3.81	25.70 ± 2.63	0.2656
Karyotype (*n*, %)				0.1212
Nonmosaic 47,XYY	32 (88.89)	28 (93.33)	4 (66.67)	
Mosaic 47,XYY/46,XY	4 (11.11)	2 (6.67)	2 (33.33)	
Basal sperm level (*n*, %)				0.5156
Normozoospermia	12 (33.33)	10 (33.33)	2 (33.33)	
Oligoasthenozoospermia	7 (19.44)	6 (20.00)	1 (16.67)	
Severe oligoasthenozoospermia	12 (33.33)	11 (36.67)	1 (16.67)	
Azoospermia	5 (13.89)	3 (10.00)	2 (33.33)	
Source of sperm for ART (*n*, %)				0.1858
Ejaculates	31 (86.11)	27 (90.00)	4 (66.67)	
Testes	5 (13.89)	3 (10.00)	2 (33.33)	

*Note*: For couples who received more than one cycle, the baseline information was based on the records of their first cycle in our center.

Abbreviations: AMH, anti‐Mullerian hormone; ART, assisted reproductive technology; BMI, body mass index; FSH, follicle stimulating hormone; ICSI, intracytoplasmic sperm injection; IVF, in vitro fertilization; LH, luteinizing hormone; PCOM, polycystic ovarian morphology; PCOS, polycystic ovarian syndrome; PGT, preimplantation genetic testing; TSH, thyroid stimulating hormone.

^a^ Missing data were removed in the analysis.

^b^ One LH level was given as “<0.10,” calculated as 0 in the analysis.

^c^ Two progesterone levels were given as “<0.05,” calculated as 0 in the analysis.

Embryo outcomes of the included 43 oocyte retrieval cycles of patients are listed in Table 2. The average results, especially important indexes such as the 2PN fertilization rate (76.37%), cleavage rate (98.21%), good‐quality embryo rate (45.57%), and blastocyst formation rate (48.35%), were reasonable for all oocyte retrieval cycles of 47,XYY syndrome patients (couples). Of the 43 cycles, 37 were from couples planned for PGT (grouped as planned PGT cycles), whereas the rest 6 were from couples receiving IVF/ICSI (grouped as IVF/ICSI cycles) (Table 2). Interestingly, the MII rate of ICSI cycles was higher than that of planned PGT cycles (*p* = 0.013, Table 2). One couple (one cycle, 15 MII oocytes retrieved) underwent freezing–thawing of oocytes (as we have mentioned above), for whom the available MII oocytes for ICSI changed from 15 to 10 after the oocytes were thawed. Therefore, for this cycle, we used the number of MII oocytes after thawing (i.e., the real MII number for ICSI) to recalculate the MII rate of all ICSI cycles, and the results became comparable between the planned PGT cycles and ICSI cycles (*p* = 0.4141, Table 2). Moreover, the D3 good‐embryo rate and total blastocyst formation rate were significantly higher in the planned PGT cycles compared with IVF/ICSI cycles (both *p* < 0.05, Table 2).

**TABLE 2:** Embryo results of all oocyte retrieval cycles.

Variables	All	Group		*P* value
		Planned PGT	IVF/ICSI	
Oocyte retrieval cycle (*n*)	43	37	6	
Maturation of oocytes				
No. of MII oocytes^a^	11.88 ± 6.73	11.41 ± 6.01	16.25 ± 11.98 [15.00 ± 12.41]^b^	0.3646 [0.5456]^b^
MII rate(%)^a^	84.40 (487/577)	83.07 (422/508)	94.20 (65/69) [86.96 (60/69)]^b^	**0.0133** [0.4141]^b^
Fertilization				
No. of oocytes fertilized (2PN)	9.09 ± 5.80	8.68 ± 4.74	11.67 ± 10.56	0.6617
Fertilization rate (2PN) (%)	76.37 (391/512)	76.07 (321/422)	77.78 (70/90) [63.33/85.00]^c^	0.7286
No. of abnormal fertilization	0.60 ± 1.03	0.59 ± 1.01	0.67 ± 1.21	>0.9999
Abnormal fertilization rate (%)	5.08 (26/512)	5.21 (22/422)	4.44 (4/90)	>0.9999
Cleavage				
No. of fertilized oocytes that cleaved	8.93 ± 5.76	8.51 ± 4.64	11.50 ± 10.71	0.6745
Cleavage rate (%)	98.21 (384/391)	98.13 (315/321)	98.57 (69/70)	>0.9999
No. of D3 good quality embryo	4.07 ± 3.03	4.11 ± 3.10	3.83 ± 2.79	>0.9999
D3 good‐quality embryo rate (%)	45.57 (175/384)	48.25 (152/315)	33.33 (23/69)	**0.0242**
Blastocyst				
No. of blastocysts (total)	4.44 ± 3.12	4.59 ± 3.07	3.50 ± 3.56	0.3924
Total blastocyst formation rate (%)	48.35 (191/395)	51.36 (170/331)	32.81 (21/64)	**0.0066**
No. of good‐quality blastocysts	2.05 ± 2.25	2.05 ± 2.20	2.00 ± 2.76	0.6786
Good‐quality blastocyst formation rate (%)	22.28 (88/395)	22.96 (76/331)	18.75 (12/64)	0.4587
Proportion of good‐quality blastocyst (%)	46.07 (88/191)	44.71 (76/170)	57.14 (12/21)	0.2807

*Note*: Bold values: statistically significant.

Abbreviations: D3, day 3; ICSI, intracytoplasmic sperm injection; IVF, in vitro fertilization; MII, metaphase II; PGT, preimplantation genetic testing; PN, pronuclei.

^a^ MII information was only calculated in ICSI and planned PGT cycles.

^b^ Due to the freezing–thawing of oocytes in one ICSI cycle, the MII data were shown and compared before or after (in []) the freezing–thawing process.

^c^ Shown as pooled or separate IVF/ICSI rate (in [ ], respectively), the denominator of the pooled rate was (for ICSI) MII number plus (for IVF) number of oocytes used for insemination.

### 3.2 PGT results

**FIGURE 2:**
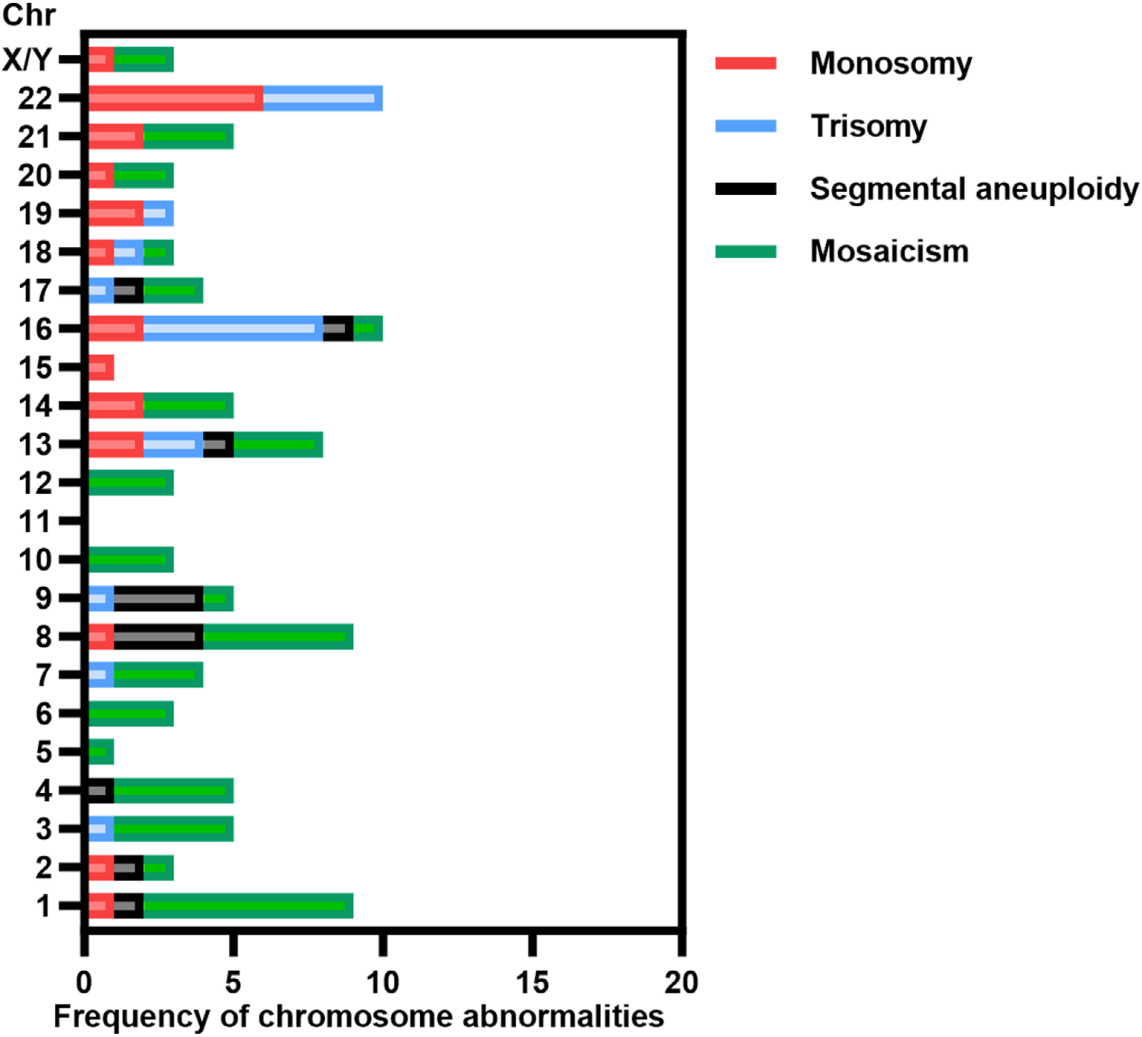
The distribution of embryo chromosomal abnormalities in PGT cycles. Chr, chromosome.

Among the 37 planned PGT cycles, two ended without the application of PGT due to the lack of available blastocysts for the test. The rest of the 35 cycles had at least one blastocyst screened by PGT. As shown in Table 3, a total of 129 embryos were tested and the euploid blastocyst rate (per cycle) was 55.04%. The aneuploid rate was not high (29.46%). Besides, 15.5% of the embryos were found to be mosaic euploidy/aneuploidy and the total mosaic rate was 22.48%. In terms of the subtypes of aneuploid blastocysts, whole chromosome aneuploidy was much more common than segmental aneuploidy (23.26% vs. 7.75%, respectively). Owing to the sex chromosome abnormality of 47,XYY syndrome, we additionally analyzed the occurrence of sex chromosome abnormalities in embryos, and it was found in only 1.55% of the embryos (two embryos). One was a monosomy of sex chromosomes (“−X”), whereas the other one was a mosaicism of both X and Y chromosomes [“+(mosaic) (X) (45%), −(mosaic) (Y) (37%)”]. To further explore whether the distribution of embryo chromosomal aberrations was influenced by paternal 47,XYY, we calculated the frequencies of the abnormalities for each chromosome in PGT embryos (Figure 2). Unexpectedly, errors were observed more often in autosomes such as 22, 16, 1, and 8 than in sex chromosomes.

**TABLE 3:** PGT results of tested embryos.

Variables	No. per PGT cycle^a^	Rate (%)
No. of blastocysts for PGT	3.69 ± 2.43	
Euploid blastocysts	2.03 ± 1.81	55.04 (71/129)
Mosaic blastocysts (total)	0.83 ± 1.12	22.48 (29/129)
Mosaic euploid/aneuploid blastocyst	0.57 ± 0.98	15.50 (20/129)
Aneuploid blastocysts (total)	1.09 ± 1.01	29.46 (38/129)
Whole aneuploid blastocysts	0.86 ± 0.97	23.26 (30/129)
Segmental aneuploid blastocysts	0.29 ± 0.46	7.75 (10/129)
Sex chromosome abnormality	0.06 ± 0.24	1.55 (2/129)

Abbreviation: PGT, preimplantation genetic testing.

^a^ Excluded the planned PGT cycles without available blastocyst for PGT.

### 3.3 Pregnancy and newborn results after embryo transfer

A total of 45 embryo transfer cycles were analyzed (Table 4). In terms of all embryo transfer cycles of the patients, both the biochemical and clinical pregnancy rates were high (80.00% and 64.44%, respectively). Over half of the ET cycles (53.33%) obtained ongoing pregnancy. The biochemical and clinical pregnancy loss rates were 19.44% and 17.24%, respectively. Importantly, a quite high live birth rate (48.89%) and infrequent preterm delivery were observed. Of the 45 embryo transfer cycles, 38 were derived from PGT cycles (grouped as PGT‐ET cycles) and 7 were from IVF/ICSI cycles (grouped as IVF/ICSI‐ET cycles). Overall, no significant difference was observed in the rates of biochemical pregnancy (84.21% vs. 57.14%, *p* = 0.1307), implantation (68.42% vs. 50.00%, *p* = 0.2785), clinical pregnancy (65.79% vs. 57.14%, *p* = 0.6860), ongoing pregnancy (52.63% vs. 57.14%, *p* > 0.9999), biochemical/clinical pregnancy loss(*p* = 0.5658 and 0.5526, respectively), live birth (50.00% vs. 42.86%, *p* > 0.9999), and preterm delivery (8.00% vs. 0.00%, *p* > 0.9999) between groups of PGT‐ET cycles and IVF/ICSI‐ET cycles (Table 4).

**TABLE 4:** Pregnancy outcomes of embryo transfer cycles.

Variables	All	Group		*p* Value
		PGT‐ET cycle	IVF/ICSI‐ET cycle	
ET cycles (*n*)	45	38	7	
Pregnancy				
Biochemical pregnancy rate (%)	80.00 (36/45)	84.21 (32/38)	57.14 (4/7)	0.1307
Implantation rate (%)	64.58 (31/48)	68.42 (26/38)	50.00 (5/10)	0.2785
Clinical pregnancy rate (%)	64.44 (29/45)	65.79 (25/38)	57.14 (4/7)	0.6860
Ongoing pregnancy rate (%)^a^	53.33 (24/45)	52.63 (20/38)	57.14 (4/7)	>0.9999
Pregnancy loss				
Biochemical pregnancy loss rate (%)	19.44 (7/36)	21.88 (7/32)	0.00 (0/4)	0.5658
Clinical pregnancy loss rate (%)^a^, ^b^	17.24 (5/29)	16.00 (4/25)	25.00 (1/4)	0.5526
Birth				
Live birth rate (%)^a^, ^b^	48.89 (22/45)	50.00 (19/38)	42.86 (3/7)	>0.9999
Preterm delivery rate (%)	6.90 (2/29)	8.00 (2/25)	0.00 (0/4)	>0.9999

Abbreviations: ET, embryo transfer; ICSI, intracytoplasmic sperm injection; IVF, in vitro fertilization; PGT, preimplantation genetic testing.

^a^ One clinical pregnancy was at 8w+ by the time of last follow‐up, so it was not counted in ongoing pregnancy, pregnancy loss, or live birth.

^b^ Two cycles with ongoing pregnancy have not yet delivered by the time of last follow‐up, thuswere not counted in pregnancy loss or live birth.

As far as neonates were concerned, totally 24 newborns were recorded by the time of the last follow‐up (Jan 2024). Normal average gestational age (38.63 weeks) and birth weight (3384 g) were obtained in the newborns (Table 5). Unexpectedly, although the rates of low birth weight (8.33%) and macrosomia (8.33%) were both low, a quite high rate of congenital anomaly (16.67%) was observed among all live newborns. Further, by comparing the neonates from PGT (named PGT‐conceived group) with neonates from IVF/ICSI (named IVF/ICSI‐conceived group), we found the gestational age (38.51 vs. 39.48 weeks, *p* = 0.8187), birth weight (3353 vs. 3597 g, *p* = 0.6877), low birth weight rate (9.52% vs. 0.00%, *p* > 0.9999), macrosomia rate (4.76% vs. 33.33%, *p* = 0.2391), male rate (61.90% vs. 33.33%, *p* = 0.5504) as well as the rate of congenital anomaly (19.05% vs. 0.00%, *p* > 0.9999) were all comparable between the two groups.

**TABLE 5:** Neonatal outcomes of the delivered newborns.

Variables	All	Group		*p* Value
		PGT‐conceived	IVF/ICSI‐conceived	
Neonates (*n*)	24	21	3	
Gestational age (weeks)	38.63 ± 2.17	38.51 ± 2.28	39.48 ± 0.84	0.8187
Birth weight (g)	3384 ± 610.33	3353 ± 623.30	3597 ± 566.60	0.6877
Low birth weight (%)	8.33 (2/24)	9.52 (2/21)	0.00 (0/3)	>0.9999
Macrosomia (%)	8.33 (2/24)	4.76 (1/21)	33.33 (1/3)	0.2391
Gender (male, %)	58.33 (14/24)	61.90 (13/21)	33.33 (1/3)	0.5504
Congenital anomaly (%)	16.67 (4/24)	19.05 (4/21)	0.00 (0/3)	>0.9999

Abbreviations: ICSI, intracytoplasmic sperm injection; IVF, in vitro fertilization; PGT, preimplantation genetic testing; PGT, preimplantation genetic testing.

## 4 DISCUSSION

Due to the rarity of 47,XYY syndrome, few reports focused on its ART results, especially PGT results. Besides, there has been no report to date discussing the necessity for infertile 47,XYY syndrome patients to conduct PGT by directly comparing the pregnancy and neonatal outcomes between PGT and conventional IVF/ICSI of such patients. In the current study, we showed the NGS‐based PGT results of 47,XYY patients (couples) and for the first time revealed the resemblance of the pregnancy and newborn outcomes between PGT and IVF/ICSI for such patients.

The clinical phenotypes of 47,XYY syndrome varied among patients, but some commonly observed symptoms were reported. For example, many studies pointed out that patients with 47,XYY syndrome were suffering from behavioral issues,^44^, ^45^, ^46^ and the patients were likely to have delayed development of motor activities.^47^, ^48^ The most typical phenotype of 47,XYY syndrome was the tall stature of patients, which was found in both adults^49^ and children.^50^ A similar phenotype was also observed in our patient cohort, as the average height was over 181 cm, far higher than the average height of Chinese male.^51^ Interestingly, the patients had a slightly higher‐than‐standard BMI (average 26.82), which might be attributed to the trend toward central adiposity of 47,XYY syndrome patients.^50^ In terms of the incidence rate of 47,XYY, a range from 0.0141% to 0.11% of the born boys was reported by different studies,^5^, ^50^, ^52^ which was quite low. Considering the fact that only part of the 47,XYY syndrome patients received ART,^53^ the readers might understand then why there was a limited number of patients (couples) included in our study. The fertility of male patients with 47,XYY syndrome had been widely discussed. Although one previous study claimed that these patients were generally fertile,^54^ other studies found that a large number of such patients might have decreased fertility potential.^13^, ^23^ In terms of sperm quality of 47,XYY patients, we found that their baseline semen varied from normozoospermia to azoospermia, which was in agreement with Borjian's and Zhang's reports.^13^, ^14^ Notably, one‐third of our patients exhibited rather normal sperm quality, and for most of these patients (couples), their ART treatments were largely attributed to female issues such as the tubal factor or diminished ovarian reserve. As for other parameters of spermatogenic function, the levels of follicle‐stimulating hormone (FSH) were only recorded in 15 of the included 36 patients, and the average FSH level was 11.28 IU/L(data not shown). The FSH level seemed to be slightly upregulated compared with males with normal spermatogenesis.^55^ Such upregulation was in line with the result of a previous study^14^ but was not found by Schiavi et al.^56^ This contradiction was presumably because Zhang's study^14^ and our study recruited 47,XYY patients at reproductive center who were seeking for treatments of infertility, thus, spermatogenic dysfunctional patients with higher FSH were more likely to be enrolled. While Schiavi's study^56^ was population‐based, which might reflect the real‐world condition.

The first highlight of this study was that we offered the NGS‐based PGT results of 47,XYY syndrome patients and exhibited the ploidy of embryos and the distribution of embryo chromosomal abnormalities in detail. Previously, Xu et al. reported the FISH‐based PGT results of 47,XYY males.^17^ However, the FISH test for PGT was mainly used in around 2010^57^ and only offered limited and blurry information of the chromosomes of embryos. With the development of sequencing technologies, the PGT has evolved to the NGS era.^58^ We found that the euploid rate of paternal 47,XYY syndrome couples was 55.04%, which was slightly lower than that reported by Yan (69.8%)^59^ but higher than that reported by Munné (43.1%)^60^ or La Marca (33.6%)^61^ in general populations. It was also lower than the average euploid rate of blastocysts based on donor oocytes (average: 64.57%).^62^ Another previous study reported the euploid rate of male patients with impaired sperm quality (regardless of paternal karyotype) was 67.5%, which was also higher than our result in 47,XYY males.^63^ Further, the euploid rate for 47,XYY patients was lower than that of 47,XXY patients (64.4%)^26^ but higher than that of males with chromosomal inversion carriers (46.1%).^33^ Interestingly, the mosaic embryo rate (referring to mosaic euploidy/aneuploidy) of embryos of 47,XYY patients seemed to be higher than that of the general population^59^, ^60^ but lower than that of 47,XXY or males with chromosomal inversion.^26^, ^33^ It is worth mentioning that one couple (one oocyte retrieval‐PGT‐FET cycle) chose to transfer a mosaic (day 5) 4AA blastocyst [karyotype: del(mosaic) (3) (q25.31q29) (41.00 Mb) (30%)] after full consultation with the genetic counseling clinic as well as the attending physician due to the lack of euploid embryos after PGT and successfully gave live birth to a boy. This suggested that, for 47,XYY syndrome patients, the transfer of mosaic embryos might result in viable and genetically normal live births, giving more chances to couples without euploid embryos. But such a decision should be very cautious and prenatal diagnosis should be offered.^64^ Surprisingly, an extremely low rate of sex chromosome abnormalities was observed in our PGT results of 47,XYY patients, much lower than that of Klinefelter syndrome patients^26^ and even much lower than that of FISH‐based PGT results of 47,XYY patients.^17^ Considering the definite superiority and more accuracy of NGS than FISH,^57^ we think that the real sex chromosome abnormality rate for 47,XYY syndrome patients' embryos was actually low.

In recent years, the necessity of PGT for several types of chromosome abnormalities, such as males with Klinefelter syndrome, women with X chromosome abnormalities or chromosomal inversion carriers, has been challenged.^26^, ^33^, ^65^ Herein, we thought that PGT was not a preferred recommendation for 47,XYY syndrome patients due to the following two reasons. First, comparable results of both pregnancy outcomes (including biochemical/clinical/ongoing pregnancy, biochemical/clinical pregnancy loss, implantation rate, live birth, and preterm delivery) and neonatal outcomes (including gestational age, birth weight, low birth weight rate, macrosomia rate, newborn gender, and congenital anomaly) were observed for males with 47,XYY syndrome whether choosing PGT or using morphology‐based embryo selection in IVF/ICSI. These phenomena indicated that although there were some extent of aneuploidies in embryos of 47,XYY patients, the PGT‐based embryo selection did not improve any pregnancy or newborn outcomes. Second, as a form of abnormalities of sex chromosomes, the 47,XYY patients had a much lower frequency of abnormalities of sex chromosomes in their embryos than the autosomal chromosomes. Interestingly, a similar phenomenon was also observed in women with X abnormalities,^65^ suggesting that the abnormal number of Y for males or the abnormal number of X for females did not have a large impact on the sex chromosomes of the offspring. By reanalyzing the PGT results of the 35 cycles of 47,XYY patients, we found the most common embryo chromosome abnormalities were observed on autosomes 16 and 22, followed by 1 and 8, which was partially similar to the results of PGT women suffering from advanced age or recurrent pregnancy loss.^66^ But abnormal chromosomes 1/8 were more frequently observed in embryos from 47,XYY patients than in embryos from general populations.^67^, ^68^ Notably, one embryo with sex chromosome abnormality was “+(mosaic) (X) (45%), −(mosaic) (Y) (37%)”. This abnormality belongs to sex chromosomal mosaicism. According to previous studies, the occurrence of mosaic embryos is primarily due to the errors in chromosome segregation in the process of the mitosis of a normal diploid zygote, and a relatively rare situation involves meiotic errors in gametes, followed by monosomy/trisomy rescue after fertilization.^69^, ^70^ Therefore, there is rather limited possibility for the case having direct correlation with paternal 47,XYY karyotype. Consequently, there was no obvious impact on sex chromosome abnormalities of embryos given by paternal 47,XYY syndrome and no apparent difference in ART outcomes brought by PGT for 47,XYY patients, thus minimizing the necessity of PGT for these patients. However, the underlying mechanisms of the enhancement of the frequency of chromosome 1/8 abnormalities remained to be uncovered.

Some limitations of the current study still need to be mentioned. First, this is only a pilot study, and due to the rather low morbidity of (nonmosaic and mosaic) 47,XYY syndrome and the even lower chances for them to seek ART treatment, the sample size of this study is limited. Second, in order to maximize the available data for analyzing, we enrolled all oocyte retrieval cycles and all embryo transfer cycles that met the criteria. Therefore, for some couples, more than one cycle was analyzed in parallel. However, different cycles of the same couple were not totally independent.^71^ Therefore, multicenter studies based on the results of the first cycle of patients should be carried out in order to balance the data size and the independence of data. Last but not least, the retrospective design of the study might lead to selection bias. Hence, this topic warrants a prospective study for more reliable results.

In conclusion, the current pilot study gave a depiction of the NGS‐based PGT results showing the ploidy status as well as the distribution of chromosomal aberrations of embryos from nonmosaic and mosaic 47,XYY patients (couples) seeking ART treatments, and a quite low rate of sex chromosome abnormalities was observed. More importantly, the results exhibited comparable pregnancy and newborn outcomes for 47,XYY patients (couples) between PGT‐ET and IVF/ICSI‐ET cycles. Taken together, PGT might not be necessary to be conducted for infertile males with 47,XYY syndrome unless there are other indications. Studies with large populations are in demand to confirm our results.

## FUNDING INFORMATION

This work is supported by the National Key R&D Program of China (2023YFC2705503), the Shanghai Science and Technology Innovation Action Plan Medical Innovation Research Project (24Y12800700, sub‐project 24Y12800703), the National Natural Science Foundation of China (81871199), the Natural Science Foundation of Shanghai (24ZR1444200), the Renji Hospital Crosswise Project (RJKY24‐005), the Three‐Year Action Plan for Strengthening the Construction of the Public Health System in Shanghai (GWVI‐11.2‐YQ21), and the Shanghai Commission of Science and Technology (20DZ2270900).

## CONFLICT OF INTEREST STATEMENT

The authors declare no conflicts of interest.

## ETHICS STATEMENT

This study was approved by Shanghai Jiao Tong University School of Medicine, Renji Hospital Ethics Committee (LY2024‐117‐B). The exemption of informed consent of patients was also approved by the Ethics Committee due to the retrospective design and anonymous data analysis.
